# Glass/steel/clay interactions in a simulated radioactive waste geological disposal system

**DOI:** 10.1038/s41598-023-47578-8

**Published:** 2023-11-21

**Authors:** I. Tolnai, J. Osan, O. Czompoly, A. Sulyok, M. Fabian

**Affiliations:** HUN-REN Centre for Energy Research, Konkoly Thege St. 29-33., Budapest, 1121 Hungary

**Keywords:** Environmental sciences, Chemistry

## Abstract

Deep geological storage is the accepted solution for the final disposal of high-level radioactive waste therefore, it is necessary to study the host rock of the planned Hungarian waste repository and the materials involved in the engineered barriers. The main goal was to understand the characteristics and stability of the glass/steel/claystone system, from the structural properties of the vitrified waste (borosilicate glasses) to the clay response in the repository. Repository conditions were applied during the experiments to understand the chemical evolution of the system. A triplicate setup was kept at 80 °C for 3, 7 and 12 months and post-mortem characterization was performed. No alteration products were observed with scanning electron microscopy energy dispersive X-ray spectroscopy measurements on the surface of the glass and Fe or in the clay after the end of the experimental period. Based on the elemental analysis of the liquid phase, the released amount of B, K, Si and Na increased, while that of Ca and Mg decreased compared to the baseline. The concentrations of Cl^−^ and SO_4_^2−^ did not change significantly. Ca- and Mg-silicate precipitation was observed by X-ray photoelectron spectroscopy at the surface range of the borosilicate glasses because of the synthetic porewater treatment.

## Introduction

Nuclear energy is one of the safest and cleanest forms of electricity generation. The Paks Nuclear Power Plant covers almost half (~ 46%) of the electricity production in Hungary, similarly high values can be observed in other countries with nuclear power plants^1^. To achieve sustainability, a great significance must be placed on the treatment and disposal of high-level radioactive waste (HLW) generated during normal operation^2^. HLW primarily includes the spent fuel (SF), as well as the waste generated by the technology during reprocessing of SF^3^. SF rods are first sent to storage pools for short-term cooling, then transferred to wet or dry storage facilities where these await either reprocessing or deep geological disposal^4^. The multi-barrier system is responsible for long-term safe storage by isolating the radioactive waste from the biosphere in deep geological repositories. Achieving this goal requires both natural geological barriers and engineered barrier system (EBS) with complementary safety functions, creating a robust system to enhance confidence in the protection that will be provided. The EBS itself comprises a variety of sub-systems such as the waste form (radioactive material immobilized in a host material), a corrosion resistant and mechanically stable container, a buffer/sealing system, and plugs. The EBS must be designed so that it will work with the natural barriers to meet the regulatory limits^5,6^. A common practice to handle and treat HLW materials is to embed them in a suitable host matrix through the process of vitrification. Glass matrices are often used for this purpose being due to their excellent chemical and mechanical parameters, their thermodynamic stability and radiation resistance are also adequate^7–9^. The vitrified HLW material is placed in a metal canister, which in most cases is made of carbon steel or stainless steel. The choice of its grade largely depends on the type of the material to be stored as well as the undesirable effects caused by the geological environment considering the goal is to ensure the containment of the radioactive material for more than 100,000 years. The buffer/sealing materials are responsible for the stability of the deep geological repository through providing desired mechanical, chemical, hydro-mechanical and thermal conditions and are characterized by low permeability and diffusivity and ensure long term retardation^6,10^.

The aim of our work was to study the chemical evolution of a glass/steel/clay system held under conditions like those that could be predicted in a deep geological repository. Long-term exposure to repository conditions could result in significant alterations on EBS materials during the service life. The host media can be source of oxygen, reactive ions and other species that can cause significant alterations to the system during its operation. The effects of radiation, temperature and mechanical stress must also be considered when various forms of degradation are calculated^10–12^.

Scale model systems were assembled in such a way that close to real conditions were provided. Our model EBS comprises borosilicate glass; modeling the waste matrix, iron; modeling the steel canister and claystone; modeling the low permeability buffer. The main goal was to understand characteristics, applicability, and stability of the whole system, from the structural properties of the vitrified waste to the clay response in the repository. Here, we demonstrate the characterization of a borosilicate glass/iron/clay model system using scanning electron microscopy energy dispersive X-ray spectroscopy (SEM/EDX) and X-ray photoelectron spectroscopy (XPS). The identifications and compositions of these materials provide information on the iron and glass alteration in function of applied conditions. With the Inductively Coupled Plasma Optical Emission Spectroscopy (ICP-OES) and Ion Chromatography (IC) we predict the chemical durability of the system, thanks to the obtained leachates at all stages of the experiments.

## Design of the experiment

### Materials

The composition of the 5-component sodium borosilicate glassy specimen was 55 mol%SiO_2_–10 mol%B_2_O_3_–25 mol%Na_2_O–5 mol%BaO–5 mol%ZrO_2_, with a density of 2.67 g/cm^3^. Synthesis and basic structural properties of the applied borosilicate glass was presented in an earlier study^13^.

Fe flakes (99%, from Acros Organics, CAS Nr. 7439-89-6) of 50–68 µm particle diameter fraction was used for the experiment. The size range was based on the consideration that the size of all three components should be commensurable during the experiments.

Boda Claystone Formation (BCF) is considered as the potential host rock system of the HLW repository in Hungary. The Boda Block of BCF forms an anticline structure. In this block the maximum thickness of the BCF varies between 700 and 1000 m in the central region. During the sedimentation catagenetic stage was finally reached under high temperature (200–250 °C) and pressure (120–150 MPa). Six main rock types of BCF can be defined based on mineralogical, geochemical, and textural considerations: albitic claystone, albitolite, “true” siltstone, dolomite interbeddings, sandstone and conglomerata. The BCF has the following parameters: porosity (%): min: 0.6-max: 1.4; hydraulic conductivity (m/s): 10^–11^–10^–13^; solid density (kg/m^3^): 2300–2700^14,15^. Since albitic claystone is the most dominant rock type of the formation^16^, a representative section of a recent drilling core called BAF-2 was selected for the present study. Table 1 contains its mineralogical composition.

**Table 1:** Mineralogical composition of the albitic claystone section of the BAF-2 drilling core of BCF.

Mineral composition	(wt%)
Vermiculite	2
Illite	24
Chlorite	6
Quartz	7
Albite	43
K-feldspar	< LOD
Calcite	5
Dolomite	8
Hematite	6

During the experiments the crushed BCF claystone was steeped with synthetic porewater resulting in a saturation of 100%. The initial BCF porewater considered in the experiment was a modeled synthetic porewater. The calculations of the Boda porewater chemistry were performed with the geochemical speciation code MINSORB and the Nagra/PSI 01/01 thermodynamic database^17,18^. The chemical composition of the Boda porewater was calculated at fixed *p*_*CO2*_ (= 10^–3.5^ bar)/pH (= 8.0) i.e., in equilibrium with atmospheric p_CO2_, and under the constraint of calcite, dolomite and quartz saturation. Na^+^ or Cl^−^ concentration was adjusted to maintain charge neutrality, while Ca, Mg, Si and C(IV) concentrations were defined over the above boundary conditions^19^. The synthetic Boda porewater (SBPW) composition is given in Table 2.

**Table 2:** Chemical composition of synthetic Boda porewater (SBPW).

Element, ion	Concentration (mol/L)
Na	1.7 × 10^–2^
K	1.8 × 10^–4^
Mg	2.3 × 10^–3^
Ca	3.1 × 10^–3^
Sr	1.5 × 10^–5^
Cl^−^	2.3 × 10^–2^
SO_4_^2−^	1.9 × 10^–3^
HCO_3_/CO_3_	6.1 × 10^–4^
Other parameters	
Ionic strength (mol/L)	3.3 × 10^–2^
pH	8.1
Eh (mV)	− 300

### Experimental setup

**Figure 1:**
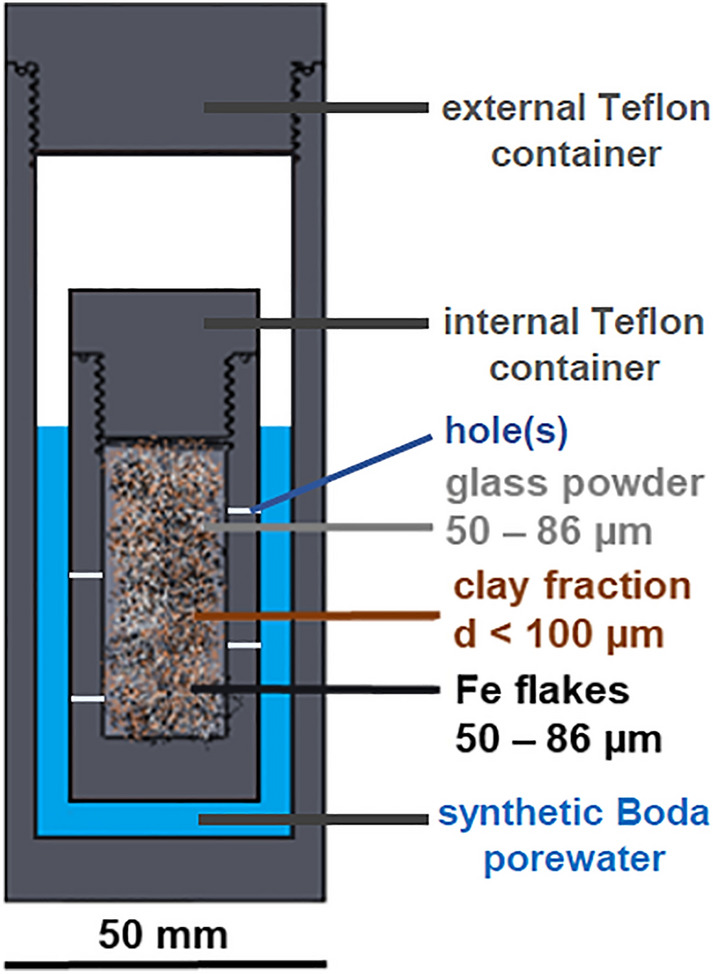
Cross-section view of the experimental setup indicating the different materials. Filled inner containers with the glass/steel/clay mixture and the external containers with the assembled inner container.

To get information on the effect of the long-term exposure a scaled-down model system was assembled close to disposal conditions to understand the chemical evolution of the contacting materials, and stability of the system from the structural and dissolution properties of the modeled vitrified waste to the clay response in the repository. Our experiment was carried out with three pieces of such glass/steel/clay model setup as shown in Fig. 1.

To provide the necessary physical conditions, the prepared setup was embedded into an external and an internal Teflon container. During the experiments all three setups were fully saturated with SBPW. The internal vessel contained the mixture of 1.4 g of powdered glass (borosilicate powder, 50–86 µm particle size, 0.083 m^2^ surface area)^13^, 0.7 g of steel (Fe powder, 50–86 µm particle size, 0.008 m^2^ surface area) and 27.9 g of claystone (crushed Boda albitic claystone, < 100 µm fraction)^19^. The conditioning of Boda claystone with the applied SBPW was carried out at room temperature. The external vessel contained the enclosed internal vessel that was surrounded by 75.36 ml of SBPW (see Fig. 1). During the static experiments, the shape of the glass grains was assumed to be cubic so that the calculated surface area to volume ratio (*SA*/*V*) was 1108 m^−1^. To ensure the continuous saturation, randomly holes with 0.7 mm diameter were drilled on the wall of the internal vessels. All the containers were filled with the same glass/steel/clay mixture and kept in an incubator at 80 °C. After 3, 7 and 12 months a container was opened for post-mortem characterization and named GFeC-3 M, GFeC-7 M and GFeC-12 M, respectively. A portion of the initial glass/steel/clay mixture was preserved at room temperature and kept dry for reference, and the used SBPW was also preserved for similar purposes.

### Sample preparation for post-mortem characterization

For morphological measurements, 5 g of samples were taken from the inner Teflon containers, and 13 mm diameter pellets were prepared using a SPECAC KBR 25.011 hydraulic press. No binder compound was needed because the applied high-pressure procedure resulted in a solid pellet.

### Glass sample preparation for X-ray photoelectron spectroscopy measurements

X-ray photoelectron spectroscopy (XPS) measurements were performed on separate glassy samples with composition of 55 mol%SiO_2_–10 mol%B_2_O_3_–25 mol%Na_2_O–5 mol%BaO–5 mol%ZrO_2_ in order to study the effect of the synthetic Boda porewater treatment at the surface range combined with the elevated temperature. For this purpose, another triplicate system was used which consisted of one Teflon container with the glass sample inside, which was immersed in SBPW, the calculated *SA*/*V* was 2000 m^−1^. The samples were heat treated the same way at 80 °C for 1, 2 and 3 months. The samples are labeled G-1 M, G-2 M and G-3 M, with the number referring to the duration of the treatment and a reference sample without treatment was used, labeled as G-Ref.

## Results and discussion

### Electron microscopy of the post-mortem samples

Scanning electron microscopy energy dispersive X-ray spectroscopy (SEM/EDX) investigations were focused on the possible crystallization and homogeneity of the glass and the nature of alteration products formed on its surface as well as the composition of the Fe and clay.

**Figure 2:**
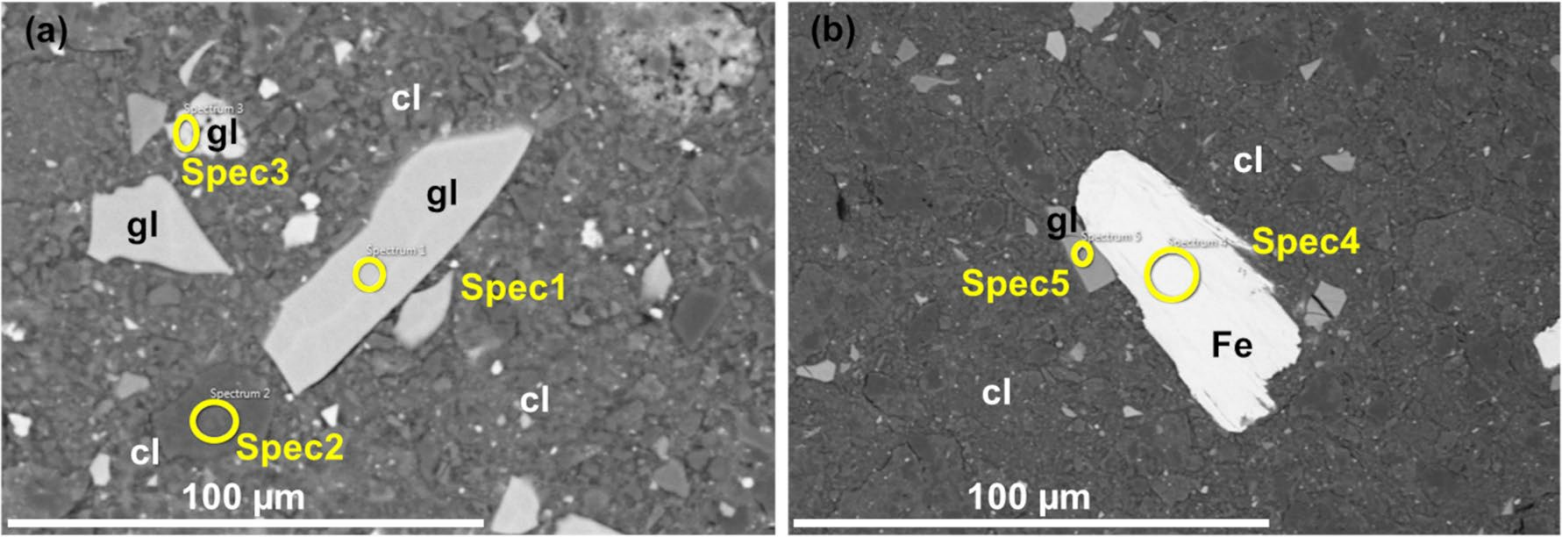
(**a, b**) SEM backscattered electron images on the GFeC-3 M sample after 3 months. Several spectra of glass particles (**a**) and Fe flakes (**b**) were measured (solids of interest are marked as gl: glass, cl: clay and Fe).

**Figure 3:**
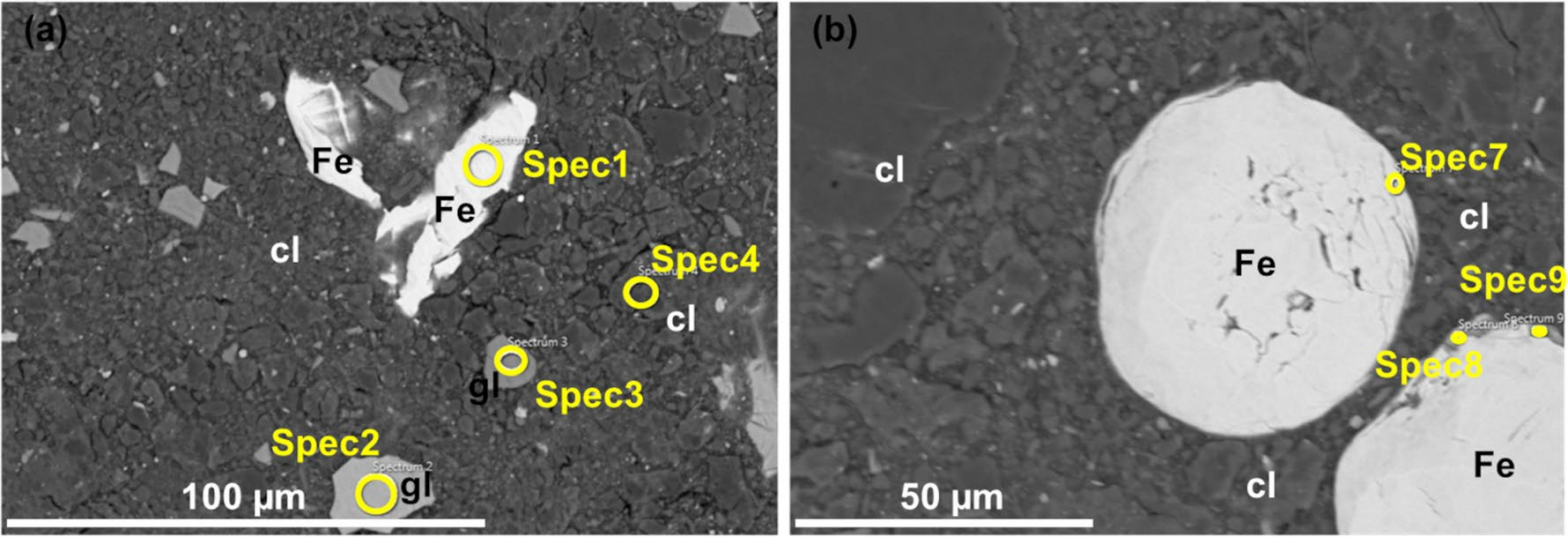
(**a, b**) SEM backscattered electron images on GFeC-7 M sample after 7 months (solids of interest are marked as gl: glass, cl: clay and Fe). Several spectra of each material (**a**) and spectras mainly focusing on Fe-clay boundaries (**b**).

**Figure 4:**
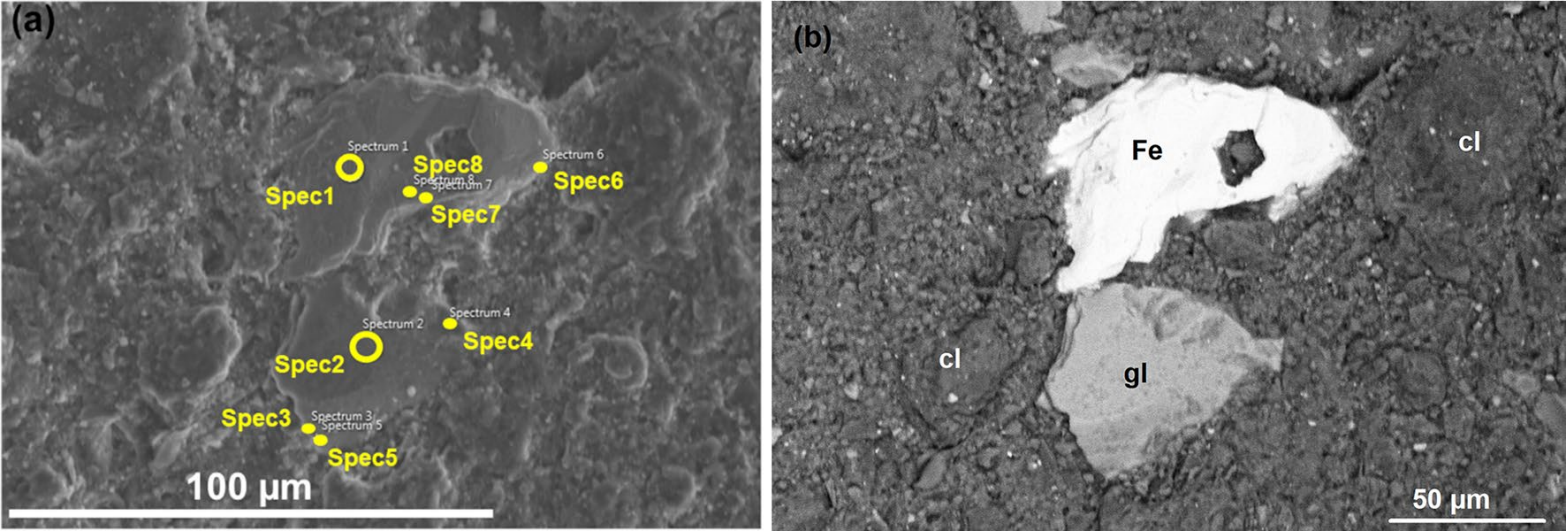
(**a, b**) SEM secondary (**a**) and backscattered (**b**) electron images on GFeC-12 M sample.

Detailed SEM images for the GFeC-3 M, GFeC-7 M and GFeC-12 M are shown in Figs. 2, 3 and 4, respectively. Based on the recorded images the structure of iron and glass did not change significantly during the experiment. Both the edges and the bulk phase remained uniform and homogeneous; no alteration layers were formed. Table 3 contains the major elemental composition for the selected positions. The elemental composition of the glass particles was rather similar in the case of the GFeC-3 M and GFeC-12 M samples meaning that the average composition within the information depth (~ 1 µm) did not change as a result of the experimental treatment. However, a slight alteration in the composition of the glass particles can be observed in the GFeC-7 M sample. A difference can be measured in O, Si, Zr and Ba content of individual glass grains as similar changes were reported in Ref.^20^. Glass phase positions for GFeC-7 M in Table 3 show a Si content of 27.3 ± 6.0%. This difference between Spec2 and Spec3 can be explained with partial hydrolysis of the glass thus allowing it to dissolve^21^. An alteration layer can be observed on the surface of the glass in similar experiments under anoxic conditions, which is typically a gel layer and its thickness increases with time^22,23^. Most gel alteration layers can be easily identified through the changes in the structure of the glass. During the dissolution of the glass, the process of hydrolysis is mostly responsible for the breaking of the Si–O–Si and Si–O–Zr bonds, therefore the number of bridging bonds decrease, while hydroxides are formed. The end of the process is that the concentration of network formers decreases. As a result of hydrolysis, the concentration of network modifiers components also changes^21,24^. The elemental composition of iron flakes did not change with time, corrosion products cannot be identified, and no sign of Fe-oxides formation was observed based on the SEM/EDX measurements.

**Table 3:** SEM/EDX results: elemental composition at selected positions after 3, 7 and 12 months (wt%).

	GFeC-3 M			GFeC-7 M					GFeC-12 M			
Element	Glass (spec1,3,5)	Fe (spec4)	Clay (spec2)	Glass (spec2,3)	Fe (spec1)	Fe edge (spec7,9)	Fe edge* (spec8)	Clay (spec4)	Glass (spec2,3, 4,5)	Fe (spec1,8)	Clay + Fe (spec6)	Fe edge (spec7)
O	44.0 ± 0.8		50.2	40.8 ± 4.0	0.4	3.5 ± 3.2	29.1	44.7	44.9 ± 2.0	1.0 ± 1.0	48.0	10.8
Na	4.4 ± 0.5		5.7	4.9 ± 0.3				2.2	4.3 ± 1.1		2.6	0.8
Mg			0.4					2.0	0.2 ± 0.1		2.4	1.0
Al	0.4 ± 0.4		11.7	0.1 ± 0.1		0.4 ± 0.4	3.7	9.2	0.9 ± 0.2		7.9	3.3
Si	27.1 ± 2.2		28.5	27.3 ± 6.0	0.1	0.5 ± 0.5	8.3	27.9	27.0 ± 0.8		21.5	6.5
K	0.3 ± 0.3		2.5	2.5 ± 2.5			0.7	4.2	0.4 ± 0.1		2.2	1.2
Ca							0.9		0.4 ± 0.2		0.7	1.3
Fe	0.7 ± 0.7	100.0			99.4	95.7 ± 4.0	57.3	6.6	0.6 ± 0.1	99.5 ± 0.7	14.2	75.1
Zr	8.3 ± 0.8			8.3 ± 1.5					9.0 ± 0.8			
Ba	15.8 ± 0.5			16.2 ± 5.9					13.1 ± 0.5		0.6	

(*) sign shows that Fe and clay are simultaneously present at the measured position, but no sign of Fe-oxide formation.

**Figure 5:**
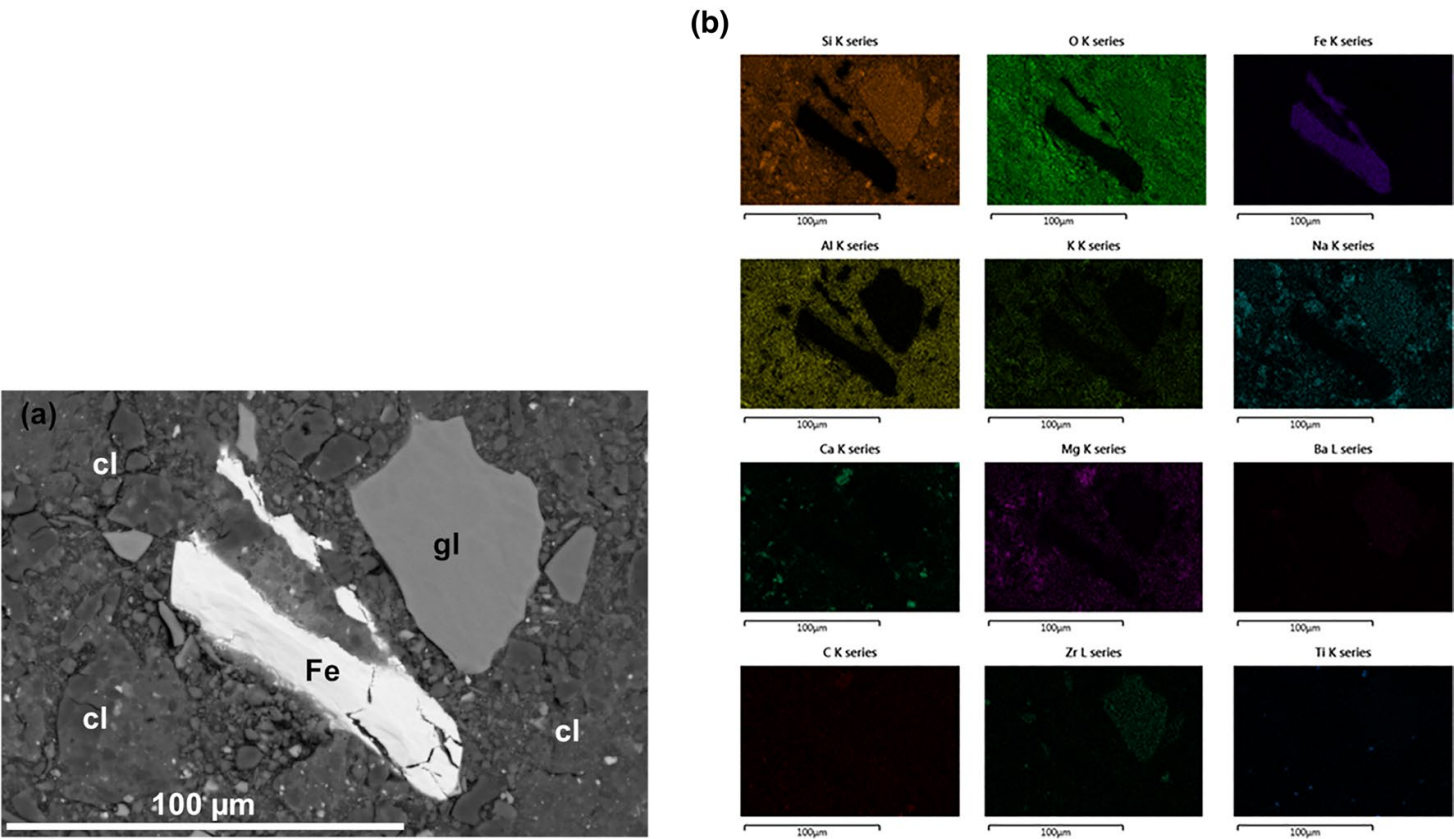
(**a, b**) SEM backscattered electron image (**a**) and the corresponding EDX elemental maps on the GFeC-7 M sample (**b**).

**Figure 6:**
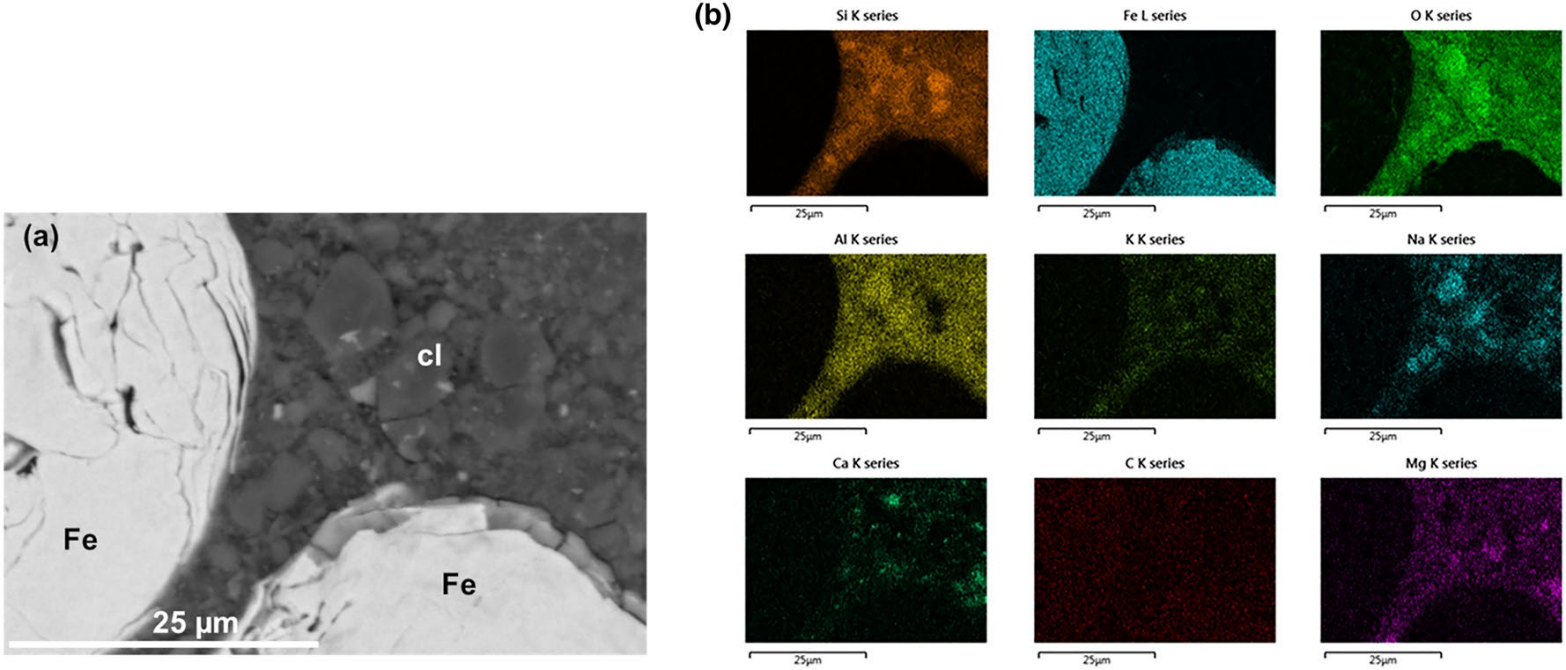
(**a, b**) Backscattered electron image (**a**) from a smaller area zoomed on Fe-clay boundaries and the corresponding elemental maps on the GFeC-7 M sample (**b**).

For further investigations, SEM/EDX elemental maps were recorded on the GFeC-7 M sample from two regions, one of 150 × 50 µm^2^ including all three types of materials (Fig. 5) and a smaller area focused on Fe-clay boundaries (Fig. 6). Elemental maps provide information on the spatial distribution of elements in the areas of interest and show the quality and quantity of elements. Based on the images, the edge and the bulk of the glass particle are uniform, based on the element map, the Fe distribution measured at the edge of the Fe particle is not uniform, but this can be explained by the uneven edges, which can be observed on the SEM backscattered image (Fig. 6). Spec8 (Fe edge* position) in Table 3 also shows that Fe and clay are simultaneously present at the measured position, no sign of Fe-oxide formation. Based on the literature data, iron corrosion products are usually present where a sufficiently large, continuous interface can be in contact with clay and glass interfaces. In such cases, in addition to Fe, there is also a significant amount of oxygen, and a small amount of Si is also present. Similar observations are reported in Ref.^25,26^.

The elemental maps recorded at 20 kV show that distribution of the Si and O components occurred homogeneously in the clay and glass phase. Grains with surplus of O and Si are present the clay. The heterogeneous character of the clay can be observed most significantly based on the distribution of K, Na, Mg and Ca.

**Figure 7:**
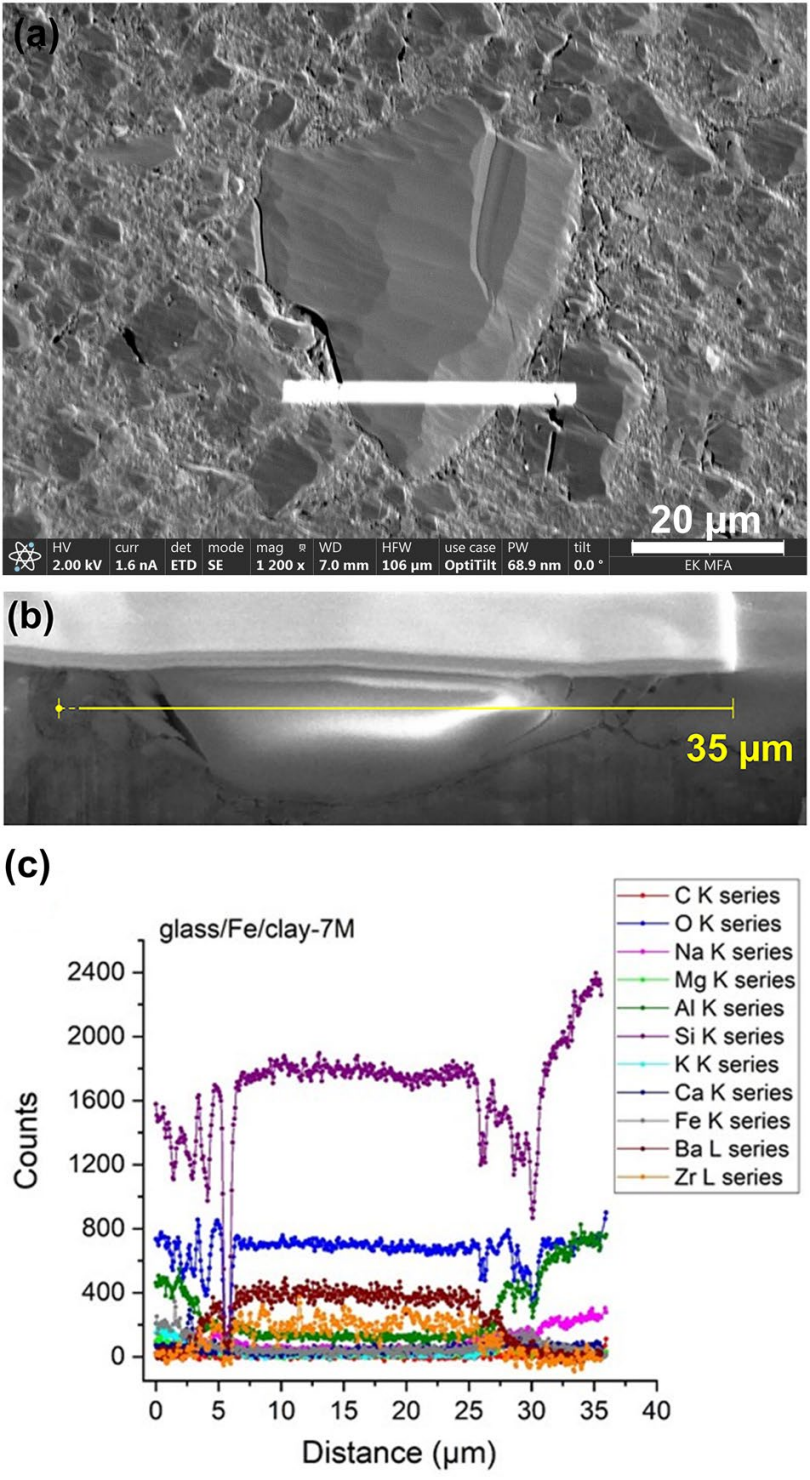
(**a, b, c**) SEM image of a focused ion-beam cross-section of the glass parts (**a**), SEM image (**b**) and the line profile of the EDS signals of the GFeC-7 M sample (**c**) measured at 20 kV.

Further SEM investigation with the combination of Focused Ion Beam (FIB) sample preparation with higher lateral resolution were conducted on the GFeC-7 M sample to elucidate the influence of the presence of Fe or clay on the alteration layer thickness of the glass particles. The SEM image and the corresponding line profile of the EDX signals are shown in Fig. 7. The glass-forming Si, O and Ba remained rather uniformly distributed in the glass particle, as the beam passes to the surface of the clay, Al can be identified from the albite in the claystone. The non-homogeneous nature of the clay can be observed by the changing distribution of Si and O. The sudden count drop can be explained by the formation of a deep crack, which is shown in Fig. 7b.

### Elemental analysis of the soaking solutions

The ICP-OES and IC results for the glass/steel/clay soaking solutions are shown in Table 4. The pH values indicate a slight increase with time: 8.12 (GFeC-3 M), 8.25 (GFeC-7 M) and 8.27 (GFeC-12 M). Higher concentrations of K, Si, B and Na can be observed in the liquid phase of all three glass/steel/clay systems compared to the initial porewater. The temperature effect could not be ruled out when experiencing elevated concentrations since the conditioning of Boda claystone with the applied synthetic porewater was carried out at room temperature. However, as our experimental setup contains not only argillaceous rocks, leaching from the other components (Fe, glass) should also be considered. Elevated Si, B and Na concentrations from the baseline can be traced back to glass content, while the increased K content came from the claystone, which contains 4.7 wt% K_2_O^27^. High Si content was measured at three months, after which the concentration decreased by an order of magnitude during the remaining course of the experiment. The concentration of Si and Na developed inversely during the experiment, while the concentration of Si decreased from the third month to the seventh month and then increased until the end of the experiment, in the case of Na, an elevated concentration can be observed in the seventh month compared to the third month, after which its concentration in the leachate slightly decreases. In contrast to these trends, in the case of B, a constant increase in concentration was observed during the experiment. Concentrations of bivalent cations including Ca and Mg decreased and the concentration of K increased one order of magnitude even after three months. A potential precipitation of potassium silicate species could be one explanation for the decreased concentration, but as a triplicate setup was applied, differences between individual containers can occur. Concentrations of Cl^−^ and SO_4_^2−^ ions in the final soaking solution are close to those of the conditioned SBPW but a slight increase was observed over time. The increase was less than 10% for Cl^−^ but more than 30% for SO_4_^2−^.

**Table 4:** ICP-OES and IC results of GFeC-3 M, GFeC-7 M and GFeC-12 M liquid phase samples. The relative error of ICP-OES and IC analysis can be estimated as 6% and 8%, respectively.

	B	Ca	K	Mg	Na	Si	Cl^−^	SO_4_^2−^
	(mg/L)							
SBPW recipe	–	125	7	57	380	–	817	182
SBPW conditioned	0.12	108	22	49	492	5.9	823	183
GFeC-3 M	27.3	59.4	232.2	28	610.7	101.8	830	234
GFeC-7 M	39.4	64.7	163.5	27.0	713.6	29.8	841	246
GFeC-12 M	45.5	64.5	162.6	23.8	673.0	40.8	891	280

Table 5 indicates the normalized mass loss and glass dissolution rate for boron for the GFeC-3 M, GFeC-7 M and GFeC-12 M samples. The obtained dissolution rates show a decreasing tendency in function of time in all sample series. B is usually assumed to provide the best measure of the extent of glass reaction as a consequence of its high solubility. The release of B occurred at an average rate of 0.0086 g/(m^2^d) until 3 months, 0.0054 g/(m^2^d) until 7 months and 0.0036 g/(m^2^d) until 12 months. The boron concentration in the GFeC-12 M soaking solution sample is 45.5 mg/L which suggests that there was no significant change in the structure of the glass during the long experimental period (in the studied borosilicate glass, B has a role of network former). Our previous leaching results on this borosilicate glass also show an order of magnitude greater normal mass loss with similar *SA/V* ratio. In that series of experiments 0.079 g/(m^2^d) normalized mass loss was measured^28^. Product Consistency Test (PCT) results on International Simple Glass under acidic to hyperalkaline conditions were calculated mass loss after 120 days for B was 0.0131 g/(m^2^d), which is consistent to our previously achieved results^29^. Results of a similar order of magnitude are also true for the borosilicate glass K-26, on which 0.0405 g/(m^2^d) normalized leaching rate was measured^30^. These low values for our glassy samples can be explained by the fact that in the present experiments the borosilicate glass did not come into direct contact with the leaching fluid as in most PCT, only in indirect contact thanks to the fully saturated clay with SBPW. It can be predicted that the saturated iron and clay mixture present in the system does not promote the chemical degradation of the glasses, therefore our glassy structure is not damaged.

**Table 5:** Normalized mass loss (*NL*_*B*_) and glass dissolution rate (*NR*_*B*_) for boron, where ${c}_{B}$ is the concentration of boron in the given soaking water, ${f}_{B}$ is the weight fraction of boron in the original borosilicate glass (unitless), $\frac{SA}{V}$ is the surface area of the final waste form divided by the volume of the leachate (m^–1^).

	*c*_B_ (mg/L)	*f*_B_	*SA*/*V* (m^−1^)	*NL*_B_ (g/m^2^)	*NR*_*B*_ (g/(m^2^d))
GFeC-3 M	27.3	0.032	1108	0.78	0.0086
GFeC-7 M	39.4	0.032	1108	1.14	0.0054
GFeC-12 M	45.5	0.032	1108	1.31	0.0036

### X-ray photoelectron spectroscopy for glass surface characterization

The composition of the prepared samples was investigated by measuring the surface in “as received” state by XPS. It determines the composition of the upper ~ 5 nm. To observe the depth dependence of composition in the near surface region, ion sputtering combined XPS analysis was applied until stable concentration was reached. Because of the insulating nature of samples, a continuous double (+ and -) charge compensation was applied to prevent electrical charging up. The surface curvature of the glass samples resulted in an uneven charging that could be reduced by decreasing analyzed spot size to 200 µm, the measured spot was at the center of the sputtered square.

**Figure 8:**
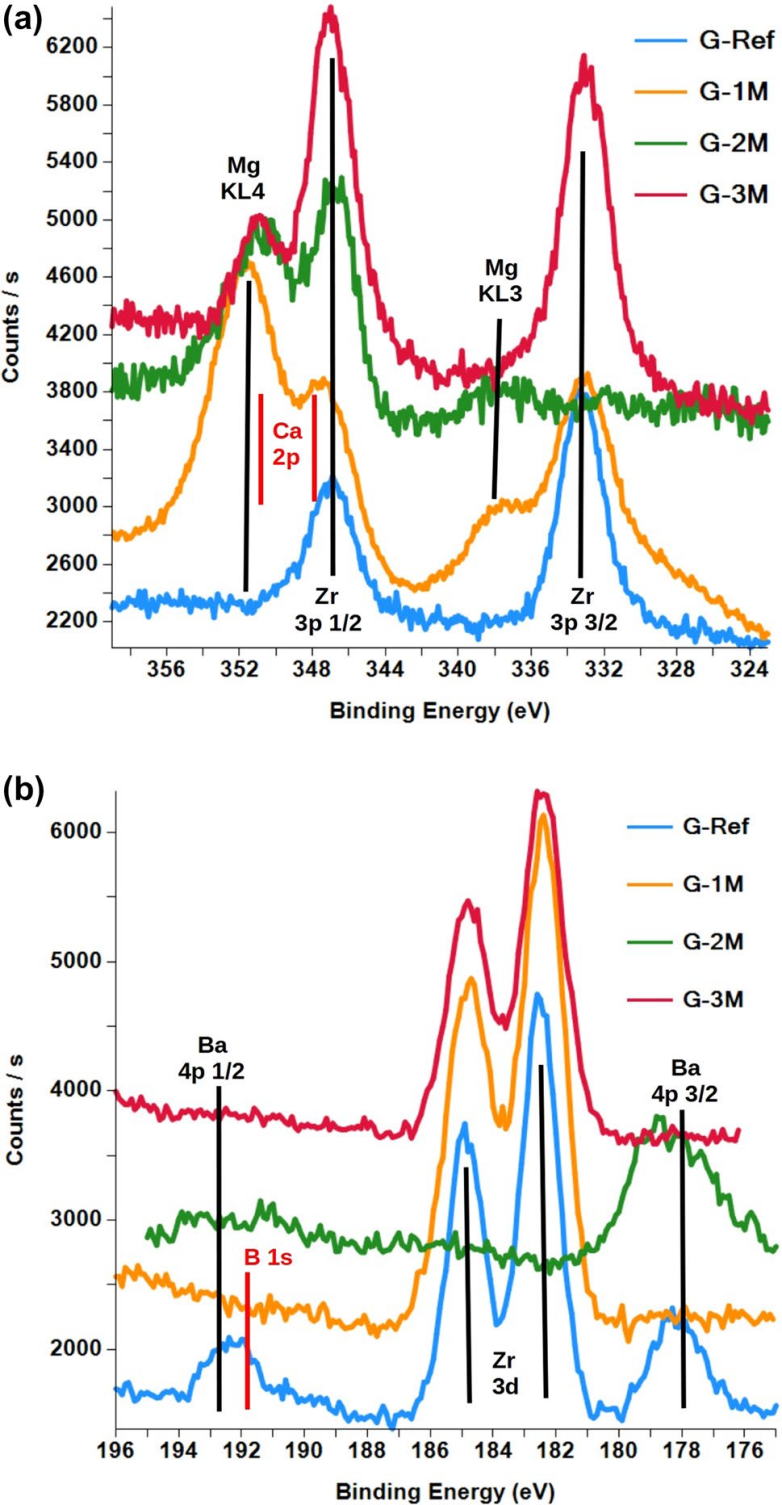
(**a, b**) The Ca region (**a**) of the XPS spectrum is shown for sample G-Ref and G-1 M (blue line—G-Ref, brown line—G-1 M) and detected XPS spectra for the Ba region (**b**) can be observed for both G-Ref and G-1 M sample (pink line—Ref, green line G-1 M).

The 3 spots of G-2 M showed a relatively larger variation of composition while the G-3 M spots showed no difference. The calculated composition (atomic %) is shown in (Table 6) (only the average value is shown where more measurements took place)**.** Table 6 shows a value where the concentration was stable, or the change of concentration with depth where it was observed. The main composition character of glass samples remained the same at the end of the treatment period. Sodium content was reduced significantly due to SBPW treatment which is consistent with our ICP-OES results, where it is also noted that after 3 months the initial conditioned SBPW’s sodium content has increased significantly, from 492 mg/L to 610.7 mg/L as seen in Table 4. A significant part of this results from the very high albite content of the BCF mineral (43 wt%), but the contribution from the dissolution of the glass system cannot be neglected^19^. Ca is absent in G-Ref sample and its presence is uncertain on G-1 M sample because of overlapping peaks, nonetheless 0.5–1% of Ca is possible. For demonstration, the Ca region of the XPS spectrum is shown in Fig. 8 for all 4 samples (G-Ref, G-1 M, G-2 M, G-3 M). G-2 M and G-3 M samples have higher Ca content, and it undoubtedly allows the separation of Ca doublet. B content was not detected in any of the samples; however, the G-Ref sample has an intense Ba peak at B 1 s energy, which prevents the observation of a small B content. Another explanation for this phenomenon is that a possible evaporation process takes place on the surface of the melt, the rate of which depends on the partial water vapor pressure in the furnace atmosphere as discussed in Ref.^31^. The detection limit of B is ~ 0.2 at% in general, however, in case of G-Ref it is estimated 0.5 at%. Detected spectra of the 4 samples presenting the B region are shown in Fig. 8. Similar leaching experiments, where the value of *SA*/*V* was the same, large release was also measured in the initial stage for boron, since leaching can most easily occur from the regions close to the surface, therefore boron cannot be detected at this depth, as it is not present there^32^. Zr was observable in each sample, however, Zr concentrations were hardly determined for G-1 M and G-2 M, since the presence of intense Mg Auger peaks prevented the correct evaluation. The smearing of Zr 3d peak is possibly due to some surface charging. Significant Mg appearance can be observed in G-1 M and G-2 M samples, which shows a decreasing trend towards G-3 M. This phenomenon can be explained by Mg–silicate precipitation, which can be realized by the presence of magnesium in the leaching solution (Table 2). This phase is limited by the glass alteration kinetics and a pH above 8 is required for the phase formation^20,33^. The pH of the leachates was 12.48, 9.64 and 8.91, for G-1 M, G-2 M and G-3 M, respectively. In addition, after 2 months, precipitation is caused not only by Mg, but also by Ca, the concentration of which also increases in the case of G-2 M and G-3 M samples as shown in Table 6, similar results are presented in Ref.^34^. The detected C signal belongs to alkane compounds which exclude the presence of CO_3_ as well.

**Table 6:** The calculated composition (atomic %) from the results of XPS measurements. The error is < 1%.

	G-Ref	G-1 M	G-2 M	G-3 M
Si	27.1	24.5	22.8	24.9
O	63.1	65.4	64.3	65.2
Na	6.0	0.75	0.4	0.36
Ca	< 0.1	< 0.5	2.1	2.2
Ba	1.1	0.15	2.3	0.09
Zr	1.5	–	–	4.0
K	2.1	0.2	< 0.1	0.4
Mg	0.1	7.2	5.8	2.9
B	< 0.5	< 0.5	< 0.5	< 0.5

**Figure 9:**
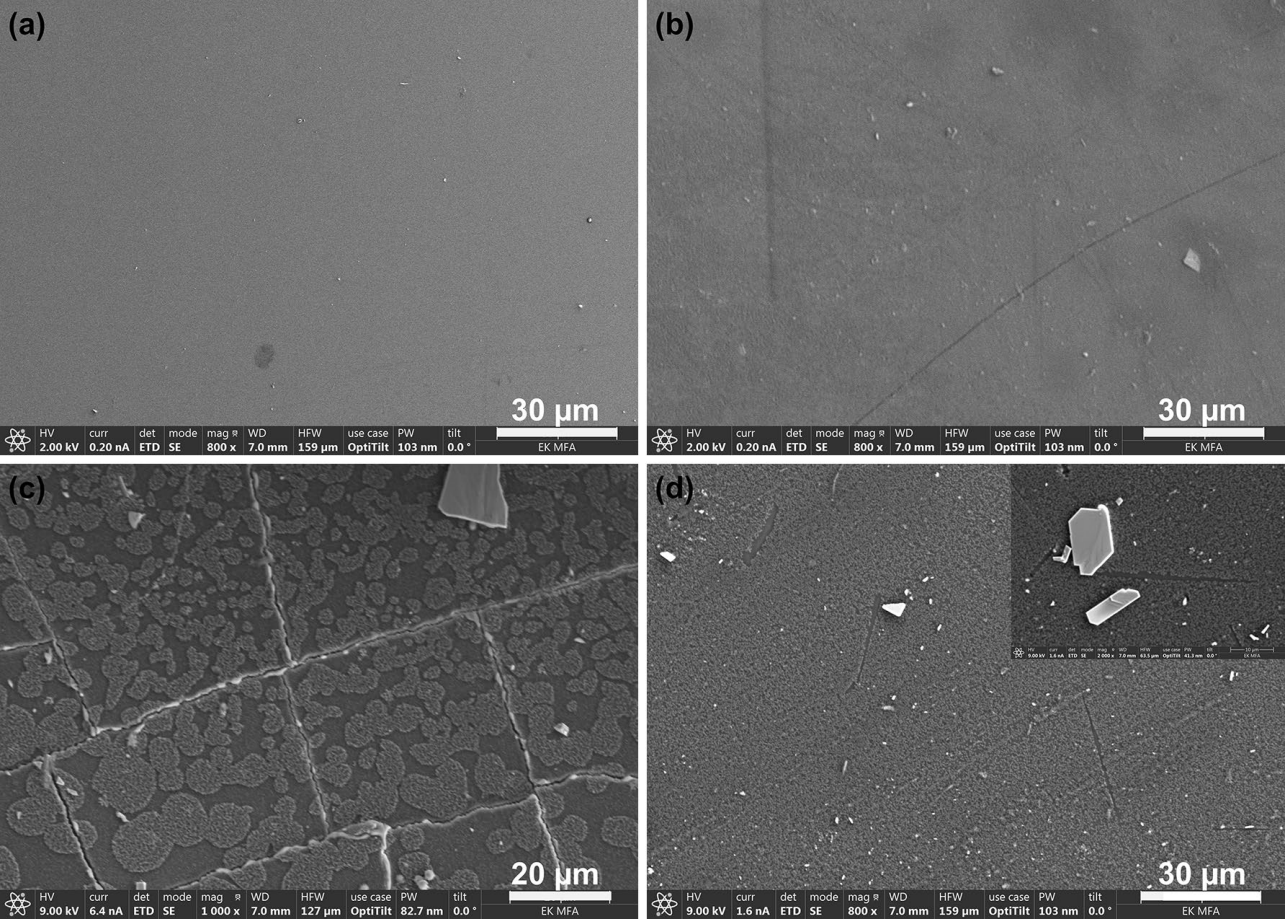
(**a, b, c, d**) Backscattered electron images at 800 × magnification on the surface of G-Ref (**a**), G-1 M (**b**), G-2 M (**c**) and G-3 M (**d**) samples.

SEM measurements were also carried out on the surface of the G-Ref, G-1 M, G-2 M and G-3 M samples, and the results are shown in Fig. 9a–d. The crystallized white agglomerates which are rich in Ca and Mg can be observed in Fig. 9b–d starting from the G-1 M sample and represent the precipitated magnesium and calcium silicate formation. Degradation of the glass surface can be observed as the experiment progresses, the most prominent change is shown on G-2 M sample, which had cracks on its surface as a result of rapid cooling after the end of the experiment. Beside the cracks, the formation of a silica-rich gel layer can be observed on the sample’s surface, started as small independent regions but at the end of the treatment this passivating layer is already continuous and homogeneous. As a result of the layer, the dissolution rate decreases as the mass transfer from the solid phase to the liquid phase will be limited^35^.

## Conclusion

We investigated the effect of the materials/conditions of the modeled engineered barrier system on each other, using promising EBS materials under conditions predictable for their lifetime. During the SEM investigations—after 3, 7 and 12 months—the glass, Fe and clay materials could be identified and investigated separately. Along the glass, the surface and borders did not vary. The main elements contained in borosilicate glass are Si, O, Na, Zr, Ba, and all of them with EDX analysis were detected after 3, 7 and 12 months. Although, no secondary phases were identified and no alteration layer has been found on the borosilicate glass samples in any case within the resolution of backscattered electron imaging at 20 keV (~ µm), the elemental composition of the investigated material changes in rather small amounts. That means the average composition within its information depth (~ 1 µm) was not altered due to experimental treatment. Supplementary surface analysis was carried out with XPS directly on the 55 mol%SiO_2_–10 mol%B_2_O_3_–25 mol%Na_2_O–5 mol%BaO–5 mol%ZrO_2_ matrix glass to observe changes and possibly secondary phases formation. The results showed that the atomic composition remained rather the same in the case of Si and O after 3 months, while Na and Ba decreased. The presence of boron within the information depth cannot be detected. The most significant finding was the precipitation of Mg and Ca on the surface of the borosilicate glass resulting from the synthetic porewater. The formation of the passive layer can be observed, which takes on an increasingly homogeneous structure as time progresses. The ICP-OES results show that the released fraction of B increased. The concentration of leached Ca and Mg among the glass-forming elements decreased which correlates with the XPS results, while the concentration of K, Si and Na increased. Ion chromatography measurement returned the initial concentration of the main ions, the concentration of Cl^−^ and SO_4_^2−^ ions did not increase significantly even during the long experiment duration.

According to the results, we can conclude that under the tested conditions (12 months, 80 °C) the used components of the engineered barrier system do not have a significant effect on each other, they individually preserve their integrity, which is the basic principle of Defense in Depth. The borosilicate glass remains a stable glass under the tested conditions, no sign of secondary phase formation and no measurable physical/chemical changes are observed. Fe flakes show no corrosion reaction. Based on our triplicate test, the minerals of BCF albitic claystone does not react with the other two engineered barriers and significantly slows down the structural transformation of the glass by delaying the alteration process.

## Methods

### Scanning electron microscopy measurements

During the Scanning Electron Microscopy with the combination of Energy Dispersive X-ray Spectroscopy (SEM/EDX) investigations, we mainly focused on the composition and the nature of alteration products formed on the Fe, within the clay and on the borosilicate glass surface. Measurements were performed using a Thermo Scientific Scios2 dual beam analytical FIB-SEM with an Oxford X-Max 20 SDD EDX attachment. The surface of the pellets was polished and covered with a thin carbon coating to improve the imaging of the samples and to avoid charge accumulation of the glass surface during electron beam investigations. Samples were measured with an accelerating voltage of 20 kV and a beam current of 1.6 nA. During the imaging of the samples, the backscattered electrons were detected, therefore the heavier elements appear brighter since these elements backscatter more strongly, in the case of GFeC-12M sample, the secondary electrons were also detected.

Focused Ion Beam (FIB) instrument was used as a sample preparation tool to mill into the surface of the material to provide cross-sectional analysis of the GFeC-7 M sample. The cross-section of the surface layers was investigated to observe the penetration depth of the porewater and to reveal any changes in the elemental composition. Before milling, a 2–3 µm thick Pt protective layer was applied to the surface using 2 kV electrons then 30 kV Ga-ions. Then a trench was milled perpendicular to the surface using the FIB beam. The imaging of the trench was performed with SEM by tilting the sample to 45° from the normal vector of the surface, all results were corrected by the effect of the tilting. The line profile of EDX signals was measured at 20 kV.

### Leaching test

Normalized release (*NL*_*B*_) and glass dissolution rate (*NR*_*B*_) were calculated for boron with the following equations based on ASTM C1285-21 protocol to gain information on the chemical evolution of the studied system and determine the chemical durability of the homogeneous glass waste form by measuring the concentration of the elements released to the synthetic porewater^36^,

1 $${NL}_{B}=\frac{{c}_{B}(sample)}{\left({f}_{B}\right)\cdot (\frac{SA}{V})}$$

2 $${NR}_{B}=\frac{{c}_{B}(sample)}{\left({f}_{B}\right)\cdot (\frac{SA}{V})\cdot (t)}$$

where ${c}_{B}$ is the concentration of boron in the given soaking water, ${f}_{B}$ is the weight fraction of boron in the original borosilicate glass (unitless), $\frac{SA}{V}$ is the surface area of the final waste form divided by the volume of the leachate (m^–1^) and $t$ is the duration of the experiment (days).

### Inductively coupled plasma optical emission spectroscopy and ion chromatography

Inductively Coupled Plasma Optical Emission Spectroscopy (ICP-OES) and Ion Chromatography (IC) investigations were performed on the aqueous solutions. 5 ml of the leachates were sampled from the liquid phase located in the outer Teflon container after 3, 7 and 12 months. The collected leachates are filtered through 0.45 µm cellulose acetate membrane. B, Ca, K, Mg, Na and Si were measured using a Perkin Elmer Avio 200 ICP-OES apparatus, all the elements were measured in radial view and Y was used as the internal standard. Ion chromatography was applied for Cl^−^ and SO_4_^−^ concentration determination. For ion chromatography a Thermo Scientific Dionex Aquion instrument was used with an A23 (2 × 250 mm^2^) column and a guard column (AS23 2 × 250 mm^2^) for Cl^−^ and SO_4_^−^ concentration determination. The applied eluent was 4.5 mM Na_2_CO_3_/0.8 mM NaHCO_3_ with electrochemical suppressing (AERS 500 Carbonate). The injection volume was 1 ml and the initial samples were diluted to 1000 times for the IC analysis.

### X-ray photoelectron spectroscopy

X-ray Photoelectron Spectroscopy is a proper technique for measuring elemental composition in the surface region. As the photoelectrons are emitted from the sample under X-ray irradiation, the elements and their valance state can be measured with high reliability within its information depth ~ 3 nm and with lower sensitivity within ~ 10 nm.

The used system was an Escalab Xi^+^ equipment of Thermo Fisher Scientific Inc.. The basic vacuum level (1 × 10^–10^ mbar) was weakened to 2 × 10^–7^ mbar during ion sputtering measurements. The ion sputtering was achieved with a 500 eV Ar^+^ beam with 45° angle of incidence scanned over 2 mm × 2 mm area. These sputtering conditions provided about 2 nm removal in 1 step. Beside of the main component lines, namely Si 2p (92 eV) and O 1 s (532 eV) several assumed minor components were observed with lines such as B 1 s (186 eV), Ca 2p (350 eV), Ba 3d (780 eV), Zr 3d (182 eV) as well as 3p (336 eV), S 2p (164 eV), K 2p (292 eV), Mg KL2 (310 eV), Na 1 s (1072 eV), C 1 s (284 eV).

Measurements started on G-Ref and G-1 M samples using 900 µm X-ray spot and was altered to 200 µm spot because of unsatisfactory charge compensation at this spot size. G-2 M and G-3 M samples were measured with 200 µm spot size. To improve the reliability of the measured composition, more location on the surface was observed as follows: 1 position for G-Ref, 4 position with 900 µm and 1 position repeated with 200 µm for G-1 M, 3 position with 200 µm for G-2 M and 3 position with 200 µm for G-3 M. Measured spectra were evaluated by determining the peak intensities. Decomposition of complex peak shapes with peak fitting algorithm were applied where necessary:

- B: B 1 s interferes with Zr 3d doublet and Ba 4p 1/2.
- Ca 2p: interferes with Zr 3p 1/2 and Mg KL4 (Auger).

Component concentration was calculated with sensitivity factors from ALTHERMO1 library by assuming homogeneous target. The XPS spectra included a carbon signal that weakened with reaching larger depth. Since the carbon signal belonged to hydrocarbon state without CO_3_, we assumed the carbon is a surface contamination and not a material component. Thus, carbon was excluded from the evaluation.

## Data Availability

The data related to the present study can be obtained from the corresponding author M. Fábián (fabian.margit@ek.hun-ren.hu) upon personal request.
